# Factors Associated with the Health-Related Quality of Life of Malaysian Young Adults Post-Pandemic

**DOI:** 10.3390/nursrep14020088

**Published:** 2024-05-10

**Authors:** Ke Xin Lee, Kia Fatt Quek, Amutha Ramadas

**Affiliations:** Jeffrey Cheah School of Medicine and Health Sciences, Monash University Malaysia, Bandar Sunway 47500, Malaysia; ke.lee@monash.edu (K.X.L.); quek.kia.fatt@monash.edu (K.F.Q.)

**Keywords:** young adults, health-related quality of life, body weight, pandemic

## Abstract

The transition from school to university brings significant adjustments in lifestyle, body weight, and living environment for young adults, potentially impacting their quality of life. Emerging evidence suggests the coronavirus disease of 2019 (COVID-19) pandemic may have exacerbated these changes. This cross-sectional study involved 361 young adults (18–25 years) from Malaysian tertiary institutions, aiming to identify factors associated with health-related quality of life (HRQOL) post-COVID-19 restrictions. Data were collected online between April and July 2022, utilizing the WHOQOL-BREF for HRQOL assessment. Participants, with a median age of 23, scored highest in the physical health domain (mean: 63.2, SD = 16.2) and lowest in psychological health (mean: 58.2, SD = 16.9). Physical health domain scores varied by ethnicity, the field of study, weight category, and prescription medicine usage; environmental health scores by ethnicity and household income; and social health scores by age. Moderate perceived stress and low perceived support were significant predictors of poor HRQOL. Across the weight categories, sleep quality, perceived stress, and support have consistently impacted the HRQOL domain scores. This study underscores the multifaceted influences on young adults’ quality of life during the transition to university, especially in post-pandemic adjustments, highlighting the importance of addressing factors such as perceived stress and support to enhance overall well-being.

## 1. Introduction

Quality of life (QOL) is a complex and multifaceted concept that includes physical health, psychological well-being, social relationships, and living conditions [1]. The World Health Organization (WHO) defined QOL as “an individual’s perception of their position in life within the cultural and value systems they inhabit, relative to their goals, expectations, standards, and concerns” [2]. In public health, health-related quality of life (HRQOL) plays an important role. Although there are varying definitions and understandings, HRQOL generally points toward perceived functioning and well-being in physical, mental, and social health domains [3,4].

HRQOL can be influenced by diverse factors, including an individual’s daily schedule and the life stages they are undergoing. Body weight status can have a significant impact on an individual’s HRQOL. Numerous studies have shown a relationship between excess body weight and decreased HRQOL in the general population and within specific age groups [5,6,7]. Obese adolescents, for example, have been shown to have the lowest total HRQOL scores, as well as physical and school scores [5].

Young adulthood is a crucial period primarily attributed to the transitional nature of this life phase. The age between 18 and 25 signifies initial experience with independent living, devoid of parental oversight, thereby instigating noteworthy alterations in lifestyle and dietary patterns and adopting detrimental habits. University lifestyle and the associated changes can impact the QOL of young adults [8]. In addition, young adults embarking on university life have been consistently shown to be predisposed to weight changes, especially weight gain [9]. Past studies have highlighted factors such as hectic schedules, breakfast omission, nocturnal snacking, stress-induced eating, the prevalence of unhealthy food options on campus, and a lack of motivation for physical activity in this demographic group [8,10,11,12]. Several studies have investigated the association between these life-changing behaviors and QOL among young adults [8,13,14,15]. However, most of the investigations were conducted on general university students without consideration of specific weight categories.

The recent coronavirus disease of 2019 (COVID-19) pandemic has resulted in significant changes in the body weight and lifestyle of these young people in Malaysia [16,17], which have also impacted their QOL. Cheah et al. [18] and Abdullah et al. [19] have explored the QOL and associated factors among Malaysian university students during the pandemic. Their studies found lower QOL as compared to pre-pandemic norms, and QOL varies according to demographic, psychological, and social aspects. Yet, there is a lack of evidence that weight changes have continued to impact the students’ lifestyle behaviors and QOL. Hypothesizing that changes in lifestyle habits and body weight will prolong post-COVID-19, our study aims to investigate the factors associated with HRQOL of young adults in tertiary institutions in the post-pandemic era.

## 2. Materials and Methods

### 2.1. Study Design

This cross-sectional study investigates the health-related quality of life (HRQOL) of young adults in Malaysia and factors associated with HRQOL during the post-COVID-19 period. Participants, comprising students from tertiary public and private institutions, were recruited using ethics board-approved recruitment flyers posted on popular social media platforms such as Facebook, Instagram, and Twitter from April to July 2022. The widespread usage of social media platforms among young adults in Malaysia made it a convenient and accessible way to reach a diverse sample.

Eligible participants were Malaysian individuals aged 18 to 25 enrolled in tertiary institutions across Malaysia, regardless of sex, socioeconomic status, or geographical location. Individuals with any inflammatory illness or medical condition that altered dietary needs, vegetarians and pregnant or breastfeeding women, were excluded.

This study received ethical approval from the Monash University Human Research Ethics Council (MUHREC) (Project ID: 30637). We use the Strengthening the Reporting of Observational Studies in Epidemiology (STROBE) checklist [20] (Supplementary Table S1) to guide the study reporting.

### 2.2. Sample Size and Study Participants

**Figure 1:**
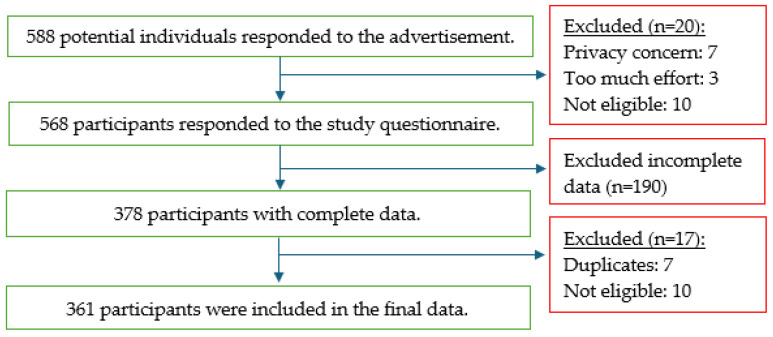
Study flow chart.

According to Kanal and Chandrasekaran [21], 15 samples for each predictor might suffice for the sample size requirement. We estimated 15 predictors to be retained in the final multinomial regression model, and a minimum number of 225 participants was required based on this estimation. A total of 588 students were invited to participate in the study, of which 568 were recruited with informed consent. After removing incomplete data, 361 participants were finally included in the study (Figure 1).

### 2.3. Measures

The data were collected using a bilingual (English and Malay) online survey form using Qualtrics (Qualtrics, Provo, UT, USA). The socio-demographic characteristics, including age, sex, marital status, occupation, ethnicity, education, household income, and family history of obesity, were recorded. Lifestyle characteristics, including smoking status, alcohol consumption, and sleeping habits were recorded. Screen time and time spent using digital devices were also included in the questionnaire. Physical activity was assessed by the International Physical Activity Questionnaire (IPAQ) [22,23], and sleeping quality was assessed using the Pittsburgh Sleep Quality Index (PSQI) [24,25]. The stress level was determined with the Perceived Stress Scale (PSS-10) [26,27], while the Multidimensional Scale of Perceived Social Support (MSPSS) [28,29] was used to determine the perceived social support. 

A dietary screener assessed participants’ overall dietary intake by measuring the frequency of consuming vegetables, fruits, cereals, fish and seafood, poultry, legumes, nuts and seeds, milk and milk products, processed meals, sugar-sweetened beverages, and water. These responses were compared against the recommendations outlined in the Malaysian Dietary Guidelines 2020 (MDG2020) [30]. For each participant, a score of one point was assigned for meeting the recommended daily intake of vegetables, fruits, cereals, fish and seafood, poultry, legumes, nuts and seeds, milk and milk products, and water. Conversely, on average, a score of one point each was given for consuming less than a serving of processed meals and sugar-sweetened beverages. These points were then totaled to obtain the MDG score, with a maximum possible score of 10. Additionally, participants’ supplement intake was recorded.

The BMI of the participants was estimated based on self-reported weight and height. The BMI was calculated using the weight (kg)/height (m^2^) formula. The BMI classification, as suggested by the Malaysian Clinical Practice Guidelines of Obesity 2004 [31], was used to classify the participants.

The HRQOL of the participants was determined using the WHOQOL-BREF questionnaire [32,33], consisting of four domains—physical health (PH), psychological health (PsyH), social relationships (SR), and environmental health (EH), in addition to two questions on overall QOL and satisfaction with life. All instruments used (IPAQ, PSS-100, MSPSS, and WHOQOL-BREF) have been validated in the Malaysian population.

### 2.4. Statistical Analysis

Frequencies and percentages are used to present categorical data, while continuous variables are expressed as mean and standard deviation (SD) or median and interquartile range (IQR). The association between categorical variables and weight categories was determined using a chi-square test. Fisher’s exact test was used as an alternative when the chi-square test assumptions were unmet. Continuous variables with skewed distribution were compared with the Kruskal–Wallis test, while one-way ANOVA was used to compare variables with normal distribution. Multinomial logistic regression analysis was performed between independent variables and overall QOL. The variables with a *p* < 0.25 in the crude analysis were incorporated into a multivariate model. All statistical analyses were conducted using IBM SPSS Statistics 29.0 with a significance level set at *p* < 0.05 (two-tailed).

## 3. Results

The majority of the participants were of normal weight (NW) (n = 172), followed by the overweight/obese (OW/OB) (n = 108) and underweight (UW) (n = 81) categories (Table 1). The median age of the study population was slightly higher in OW/OB (Md = 23, IQR = 2) compared to the other groups (*p* = 0.001). Overall, there were more females in all three weight categories, though the proportion appeared to be significantly higher in UW (87.7%) compared to other weight categories (*p* < 0.001). Ethnicity was also associated with the body weight category (*p* = 0.008), with Chinese students forming a higher proportion of participants in the UW and NW categories. A family history of obesity was significantly associated with weight categories (*p* = 0.005). The proportion of participants with a family history of obesity was highest in the OW/OB group (38.9%) compared with NW (25.0%) and UW (18.5%) groups. As anticipated, the median BMI significantly differed according to weight categories (*p* < 0.001). 

The HRQOL domain scores were compared between the demographic characteristics and the participants’ medical history (Table 2). The PH domain scored the highest mean of 63.2 (SD = 16.2), followed by the EH domain with a mean of 62.6 (SD = 15.4). The PH domain scores differed according to ethnic groups, course studied, weight category, and use of prescription medicine.

For participants of Chinese ethnicity, the PH score was significantly higher than those of another ethnicity group (mean difference, MD = 13.09, *p* = 0.027). Young adults enrolled in medicine and health sciences-related courses had higher PH domain scores than non-medical related courses (MD = 4.27, *p* = 0.026). OW/OB participants had lower PH domain scores than NW participants (MD = −6.44, *p* = 0.003), while those taking prescription medicine also had lower PH domain scores than participants without prescription medicine (MD = 6.43, *p* = 0.043). The SH domain score significantly differed according to age group, where 18–20 years had a significantly lower SH domain score than 21–23 years (MD = −8.36, *p* = 0.030). The EH was significantly lower in participants of other ethnicities than Malay (MD = −13.35, *p* = 0.021) and Chinese (MD = −12.42, *p* = 0.027) participants. 

Poor overall QOL was associated with perceived stress and support (Table 3). Those with moderate stress levels have lower odds for poor QOL (AOR = 0.11, 95% CI = 0.04–0.32, *p* < 0.001). Participants with low levels of support have significantly higher odds for poor overall QOL than those receiving a high support level (AOR = 20.83, 95% CI = 3.47–125.06, *p* < 0.001). Participants with low physical activity levels have lower odds for good overall QOL than active young adults (AOR = 0.35, 95% CI = 0.16–0.77, *p* = 0.009). Those with low-stress levels have significantly higher odds for good overall QOL than participants with high-stress levels (AOR = 9.23, 95% CI = 1.99–42.79, *p* = 0.005). Young adults with low and moderate support levels have lower odds for good QOL compared with those with high levels of perceived support (AOR = 0.19, 95% CI = 0.06–0.58, *p* = 0.003, and AOR = 0.31, 95% CI = 0.18–0.54, *p* < 0.001, respectively). 

We further correlated the QOL domain scores with independent factors according to the weight categories (Table 4). The perceived stress score was negatively correlated with all domains of QOL within the UW group. Higher Global PSQI scores indicated poor sleep quality negatively correlated with PH, PsyH, and EH domain scores. The PH domain score was positively correlated with the night sleep duration and perceived support score. The perceived support score was also positively correlated with the PsyH domain, while the duration of digital media use for entertainment was positively correlated with the EH domain score in this group. 

The association between sleep quality, stress, support scores, and QOL domains was more apparent in the NW group (Table 4). In the NW group, all QOL domains were positively correlated with support scores and negatively correlated with sleep quality and stress scores. We also found a positive correlation between household income, night sleep duration, and PH domain score. In addition, total physical activity score was positively correlated with PsyH and SH scores in this group. BMI was found to be positively correlated only with the EH score.

Among OW/OB young adults, there was a consistent negative correlation between all QOL domains and sleep quality and stress score. Support scores were positively correlated with PsyH, SH, and EH domain scores.

## 4. Discussion

This study was conducted among young adults studying in Malaysian tertiary institutions during the post-COVID-19 period. We found the PsyH domain to score the lowest among all the HRQOL dimensions (Table 2), which was supported by a past review that found college students have poorer QOL in terms of mental health [8]. Several local and international studies have reported university students’ lowest mental health domain scores during the pandemic and immediately post-pandemic, regardless of the QOL tool used [13,18,34,35]. The low scores observed in the psychological QOL domain among university students could be attributed to stressors and disruptions to students’ lives due to the restricted lifestyle during the pandemic. This includes sudden transitions to online learning, social isolation, financial concerns, and fears about the virus’s impact on health and prospects [36]. These stressors can aggravate existing mental health issues or lead to the development of new ones, such as anxiety, depression, and loneliness [37], which could reflect an overall lower PsyH score. 

Further investigation of the HRQOL domain scores showed that the sex of the study population was not associated with HRQOL, which is consistent with previous evidence [8]. Similarly, overall, QOL was not associated with the age of our study population. However, students in the 21–23 years category had significantly higher SH scores compared with the younger age category (18–20 years). It can be hypothesized that those in the older age group gradually mature and gain more confidence, which could have influenced their social well-being.

We observed disparities in QOL domain scores according to ethnicities and income groups. Students of Chinese ethnic backgrounds, for example, reported the highest PH and EH domain scores. One hypothesis could be that cultural factors associated with specific ethnicities may influence health behaviors and perceptions, impacting physical health outcomes. For example, cultural practices related to diet, exercise, and healthcare utilization may vary among different ethnic groups, potentially influencing their overall physical health [38]. We also found that the EH score was highest in students from the top 20% income category, and specifically, household income was associated with PH score among students with NW. This finding is consistent with Zhang et al.’s [39] study, which reported a positive association between higher family income and higher scores in the environmental dimension among college students. Individuals from higher-income households may have greater access to healthcare services, healthier living environments, and recreational facilities, which could contribute to better PH and EH QOL [40,41].

Apparently healthier students, characterized by NW, and those who never consumed prescription medicine, reported higher PH scores. Students with NW may be more likely to engage in healthy behaviors such as regular exercise and balanced nutrition, which can positively impact the PH QOL. Additionally, the absence of prescription medication may indicate better overall health status and fewer chronic health conditions, leading to higher self-reported PH scores. This finding aligns with the existing literature highlighting the role of lifestyle factors in promoting physical health and well-being among young adults [42].

Perceived support also emerged as an essential factor influencing QOL in young adults (Table 3). Those with poor overall QOL had low levels of support, and this association remained consistent across almost all HRQOL domains and weight categories. Alternately, those with good overall QOL had a low-moderate level of perceived support. This finding aligns with recent studies, such as Cahuas et al.’s [43] survey among college students during COVID-19, which showed social support from family to be a significant predictor of QOL. Social support is critical in buffering against stress, promoting psychological well-being, and enhancing overall QOL [44,45].

A low level of physical activity negatively influenced good overall QOL. The inverse relationship between low levels of physical activity and QOL among young adults and university students aligns with the established literature and common understanding [46]. Specifically, we demonstrated a positive correlation between physical activity and SH and PsyH domains but only among young adults with normal body weight. One potential explanation for this could be that students with normal body weight who engage in higher levels of physical activity may experience more excellent subjective health and psychological well-being due to the positive effects of exercise on mood regulation, stress reduction, and overall physical fitness [47].

Based on previous studies, such as [13] which suggested relationships between BMI status and QOL, we further explored the correlation between sociodemographic and lifestyle factors and QOL domains in specific weight categories (Table 4). Stress was one of the most important factors negatively correlated with overall QOL and all HRQOL domains across all weight categories. This finding is consistent with previous studies [48,49,50] that have highlighted the detrimental effects of stress by contributing to psychological distress, physical health problems, and impaired social functioning, thereby impacting an individual’s QOL.

In our study, poor sleep quality was found to be correlated with most QOL domains, if not all domains, across all weight categories. Past research has consistently demonstrated this relationship in the general population [51,52,53] and within specific age groups [54,55,56,57,58]. Disrupted sleep patterns can lead to fatigue, mood disturbances, and decreased cognitive function, detracting from overall QOL [59].

### Strengths and Limitations

Although this study did not impose a purposive sampling technique, it included a diverse sample of university students from Malaysian tertiary institutions, enhancing the findings’ generalizability to the broader student population. By having students from various ethnicities, socioeconomic backgrounds, and academic disciplines, this study captures a wide range of perspectives and experiences. We also adopted a multidimensional approach to assess HRQOL by examining multiple domains, including physical, psychological, and environmental health. This comprehensive assessment allows a greater understanding of the factors influencing students’ well-being.

The cross-sectional nature of the study design limits our ability to establish a causal relationship between exposure and HRQOL. The reliance on self-report measures for assessing HRQOL and exposure measures may introduce response bias, such as social desirability or recall bias. The online data collection made detailed dietary assessment difficult, leading us to resort to using a dietary screener. This tool provides us with an average estimate of participants’ dietary intake compared to the recommendations. The online data collection has also hindered more objective assessment of anthropometry measures. The psychometric properties of previously validated instruments that have been adapted for online administration are also unknown. While the diverse sample of university students was this study’s strength, there is also a potential risk of limited generalizability of the findings to all young adults in Malaysia.

## 5. Conclusions

Based on the comprehensive analysis of various factors impacting the HRQOL among young adults in Malaysian tertiary institutions during the post-COVID-19 period, it is evident that mental health emerged as a significant concern, with psychological domain scores being notably lower. Stressors related to the pandemic, such as online learning transitions and social isolation, likely contributed to this trend. Additionally, disparities in HRQOL were observed based on factors like ethnicity, income, and lifestyle behaviors. These findings underscore the importance of targeted interventions to address mental health challenges and promote holistic well-being among university students in Malaysia, particularly in the aftermath of the pandemic. Further longitudinal studies are warranted to validate these findings and inform evidence-based interventions to enhance the HRQOL of young adults.

## Figures and Tables

**Table 1:** Demographic characteristics, medical history, and anthropometry of the study population (N = 361).

		Total	Weight Category ^1^			*p*-Value
			UW	NW	OW/OB	
		(N = 361)	(n = 81)	(n = 172)	(n = 108)	
Age (years)	Median (IQR)	23 (2)	22 (1)	22 (3)	23 (2)	0.001 *
Sex	Female	276 (76.5)	71 (87.7)	136 (79.1)	69 (63.9)	<0.001 **
	Male	85 (23.5)	10 (12.3)	36 (20.9)	39 (36.1)	
Ethnicity	Malay	86 (23.8)	14 (17.3)	35 (20.3)	37 (34.3)	0.008 *
	Chinese	248 (68.7)	60 (74.1)	129 (75.0)	59 (54.6)	
	Indian	14 (3.9)	4 (4.9)	5 (2.9)	5 (4.6)	
	Other	13 (3.6)	3 (3.7)	3 (1.7)	7 (6.5)	
Marital status	Single	341 (94.5)	77 (95.1)	163 (94.8)	101 (93.5)	0.874
	Committed	20 (5.5)	4 (4.9)	9 (5.2)	7 (6.5)	
Education level	Bachelor	225 (62.3)	54 (66.7)	106 (61.6)	65 (60.2)	0.315
	Honors	90 (24.9)	22 (27.2)	43 (25.0)	25 (23.1)	
	Postgraduate	46 (12.7)	5 (6.2)	23 (13.4)	18 (16.7)	
Course	Medicine/Health sciences	43 (25.0)	28 (34.6)	43 (25.0)	25 (23.1)	0.172
	Others	129 (75.0)	53 (65.4)	129 (75.0)	83 (76.9)	
Household income (MYR)	Median (IQR)	5000 (5500)	4000 (6750)	5000 (5000)	5000 (2500)	0.421
BMI (kg/m^2^)	Median (IQR)	20.8 (4.9)	17.0 (1.6)	20.5 (2.0)	26.1 (5.5)	<0.001 **
Prescribed medicine	Yes	28 (7.8)	6 (7.4)	12 (7.0)	10 (9.3)	0.778
	No	333 (92.2)	75 (92.6)	160 (93.0)	98 (90.7)	
Family history of obesity	No	261 (72.3)	66 (81.5)	129 (75.0)	66 (61.1)	0.005 *
	Yes	100 (27.7)	15 (18.5)	43 (25.0)	42 (38.9)	

^1^ UW = underweight; NW = normal weight; OW/OB = overweight or obese. Continuous variables with skewed data distribution are presented as median (IQR), and categorical variables are presented as n (%). * significant at *p* < 0.05; ** significant at *p* < 0.001.

**Table 2:** Comparison of quality of life scores of the study population according to demographic characteristics and medical history (N = 361).

			QOL Domain ^1^							
		N	PH	*p*-Value	PsyH	*p*-Value	SH	*p*-Value	EH	*p*-Value
Total		361	63.2 (16.2)		58.2 (16.9)		58.3 (19.5)		62.6 (15.4)	
Age (years)	18–20	43	64.4 (14.9)	0.868	54.3 (17.5)	0.258	50.9 (19.9)	0.028 * ^4^	62.0 (16.2)	0.555
	21–23	220	62.9 (16.5)		58.8 (17.0)		59.4 (18.8)		64.0 (13.8)	
	24–25	98	63.2 (16.2)		58.8 (16.4)		58.3 (19.5)		62.6 (15.4)	
Sex	Female	276	62.6 (15.9)	0.184	57.7 (17.0)	0.297	58.9 (19.7)	0.255	62.3 (15.1)	0.520
	Male	85	65.2 (17.1)		59.9 (16.7)		56.2 (18.8)		63.5 (16.5)	
Ethnicity	Malay	86	62.8 (17.3)	0.036 * ^5^	59.2 (18.0)	0.736	59.1 (18.5)	0.070	64.0 (14.4)	0.013 * ^5,6^
	Chinese	248	64.1 (15.3)		58.0 (16.5)		59.0 (19.3)		63.0 (15.3)	
	Indian	14	60.7 (23.9)		59.9 (20.2)		51.3 (22.9)		56.9 (20.5)	
	Other	13	51.0 (10.8)		54.0 (13.0)		46.5 (23.0)		50.6 (14.2)	
Marital status	Single	341	63.2 (16.4)	0.985	58.1 (17.1)	0.446	58.0 (19.3)	0.197	62.6 (15.5)	0.761
	Committed	20	63.2 (13.2)		61.1 (13.3)		63.8 (21.5)		61.6 (13.9)	
Education level	Bachelor	225	62.6 (16.4)	0.537	57.8 (17.1)	0.775	57.0 (20.1)	0.252	61.5 (16.0)	0.195
	Honors	90	64.8 (16.6)		59.2 (18.0)		60.5 (18.1)		63.8 (15.6)	
	Postgraduate	46	62.7 (14.2)		58.7 (13.8)		60.4 (19.1)		65.4 (11.9)	
Course	Medicine/Health sciences	96	66.3 (15.9)	0.026 *	57.0 (18.5)	0.413	59.0 (19.1)	0.655	64.9 (15.8)	0.091
	Others	265	62.0 (16.2)		58.7 (16.3)		58.0 (19.7)		61.7 (15.3)	
Household income ^2^	B40	82	61.6 (14.9)	0.213	58.9 (16.0)	0.755	61.1 (17.0)	0.529	61.3 (14.0)	0.005 * ^7,8^
	M40	71	64.1 (21.0)		60.8 (20.4)		58.6 (23.8)		62.6 (18.9)	
	T20	15	70.1 (14.4)		61.7 (14.3)		64.6 (17.9)		75.9 (10.3)	
Weight category ^3^	UW	81	63.5 (14.9)	0.005 * ^9^	60.5 (15.4)	0.050	61.2 (18.2)	0.052	63.6 (14.7)	0.195
	NW	172	65.6 (15.9)		59.3 (17.6)		59.3 (19.6)		63.5 (14.4)	
	OW/OB	108	59.1 (16.9)		54.9 (16.5)		54.5 (19.8)		60.3 (17.4)	
Prescribed medicine	Yes	28	57.3 (17.3)	0.043 *	56.5 (19.1)	0.570	52.6 (22.2)	0.111	58.4 (15.4)	0.133
	No	333	63.7 (16.0)		58.4 (16.7)		58.8 (19.2)		62.9 (15.4)	
Family history of obesity	Yes	100	61.5 (17.6)	0.217	58.0 (15.7)	0.882	56.3 (20.1)	0.223	61.0 (15.5)	0.223
	No	261	63.8 (15.6)		58.3 (17.4)		56.1 (19.2)		63.2 (15.4)	

Data presented as mean (SD). ^1^ PH = physical health; PsyH = psychological health; SH = social health; EH = environmental health; ^2^ N = 168. B40 = bottom 40% (<MYR4850); M40 = middle 40% (MYR4850-10959); T20 = top 20% (>MYR10960); ^3^ UW = underweight; NW = normal weight; OW/OB = overweight or obese. Significant difference between; ^4^ 18–20 yr vs. 21–23 yr; ^5^ Chinese and others; ^6^ Malay and others; ^7^ B40 and T20; ^8^ M40 and T20; ^9^ NW and OW/OB as showed by post-hoc Bonferroni test. * significant at *p* < 0.05.

**Table 3:** Multinomial logistic regression between factors associated with overall quality of life of the study population (N = 361).

		Overall QOL ^1^				
	Poor			Good		
	AOR	95% CI	*p*-Value	AOR	95% CI	*p*-Value
Age	0.79	0.60–1.02	0.073	0.97	0.83–1.14	0.973
Family history of obesity						
Yes	0.47	0.15–1.46	0.192	1.11	0.64–1.93	0.718
No	1.00			1.00		
Total MDG score	0.85	0.62–1.17	0.318	1.06	0.89–1.25	0.521
Physical activity level						
Inactive	1.31	0.27–6.33	0.739	0.35	0.16–0.77	0.009 *
Minimally active	0.90	0.15–5.53	0.911	0.83	0.35–1.95	0.829
HEPA active	1.00			1.00		
Sleep quality						
Poor	3.66	0.88–15.24	0.075	0.61	0.33–1.12	0.113
Good	1.00			1.00		
Duration of night sleep	0.95	0.70–1.30	0.761	1.06	0.87–1.29	0.587
Perceived stress						
Low	0.80	0.10–6.56	0.831	9.23	1.99–42.79	0.005 *
Moderate	0.11	0.04–0.32	<0.001 **	1.26	0.49–3.27	0.628
High	1.00			1.00		
Perceived support						
Low	20.83	3.47–125.06	<0.001 **	0.19	0.06–0.58	0.003 *
Moderate	4.61	0.94–22.63	0.060	0.31	0.18–0.54	<0.001 **
High	1.00			1.00		
Duration of digital media use for social networking	1.16	0.99–1.36	0.058	1.02	0.92–1.12	0.762

AOR = adjusted odds ratio; * significant at *p* < 0.05; ** significant at *p* < 0.001. ^1^ Neutral is the reference category. Only variables with *p* < 0.25 in crude regression analysis and selected for multivariate analysis are shown.

**Table 4:** Pearson’s correlation between significant factors and quality of life domain scores of the study population according to weight categories (N = 361).

	QOL Domain ^1^			
	PH	PsyH	SH	EH
**Weight category: Underweight**				
Duration of night sleep	0.350 *			
Global PSQI score	−0.326 *	−0.339 *		−0.429 *
Perceived stress score	−0.451 *	−0.532 **	−0.425 *	−0.408 *
Perceived support score	0.412 *	0.454 *		
Duration of digital media use for entertainment				0.315 *
**Weight category: Normal weight**				
BMI				0.305 *
Household income	0.236 *			
Total MET score		0.299 *	0.412 **	
Duration of night sleep	0.260 *			
Global PSQI score	−0.476 **	−0.467 **	−0.416 **	−0.251 *
Perceived stress score	−0.681 **	−0.617 **	−0.407 **	−0.450 **
Perceived support score	0.588 **	0.589 **	0.574 **	0.561 **
**Weight category: Overweight/obese**				
Global PSQI score	−0.537 **	−0.450 **	−0.349 *	−0.378 *
Perceived stress score	−0.432 *	−0.631 **	−0.348 *	−0.404 *
Perceived support score		0.289 *	0.530 **	0.294 *

Only variables with significant correlations are shown. ^1^ PH = physical health; PsyH = psychological health; SH = social health; EH = environmental health. * Significant at *p* < 0.05; ** significant at *p* < 0.001.

## Data Availability

The data presented in this study are available on request from the corresponding author.

## Supplementary Materials

The following supporting information can be downloaded at: https://www.mdpi.com/article/10.3390/nursrep14020088/s1, Table S1: STROBE Statement.

## Public Involvement Statement

No public involvement in any aspect of this research.

## Guidelines and Standards Statement

This manuscript was drafted against the STROBE: STrengthening the Reporting of OBservational studies in Epidemiology.
